# Pathology and molecular diagnosis of classical swine fever in Mizoram

**DOI:** 10.14202/vetworld.2015.76-81

**Published:** 2015-01-24

**Authors:** David Malswamkima, T. K. Rajkhowa, Rajesh Chandra, T. K. Dutta

**Affiliations:** Department of Veterinary Pathology, College of Veterinary Sciences and Animal Husbandry, Central Agricultural University, Selesih, Aizawl, Mizoram, India

**Keywords:** classical swine fever, Mizoram, reverse transcription polymerase chain reaction

## Abstract

**Aim:**

Clinical histopathological and molecular diagnosis of classical swine fever disease in pigs of Mizoram.

**Materials and Methods:**

Totally, 31 clinically suspected pigs from 6 districts of Mizoram were examined, and clinical symptoms were recorded. Detailed post mortem examination of all the 31 dead animals was conducted, and gross changes were recorded. Tissue samples were collected for histopathological examination and molecular diagnosis. The collected tissues (tonsil, lymph nodes, spleen) were also processed for RNA extraction. Reverse transcription polymerase chain reaction (RT-PCR) was performed to detect the specific gene fragments of classical swine fever virus (CSFV).

**Results:**

Clinical examination of all the 31 suspected pigs revealed typical clinical signs of CSF. All the animals also showed typical gross and microscopic lesions of CSF. RT-PCR on tissue samples amplified the 421bp, 449bp and 735bp region of 5´NCR, non-structural protein 5B and E^rns^ gene regions of CSFV, respectively. Nested PCR for internal region of E2 gene also amplified the expected product of 271bp using PCR product of whole E2 region as template DNA.

**Conclusion:**

CSF is highly endemic disease in Mizoram. The viral strains circulating in this region are highly virulent. The disease can be diagnosed specifically using RT-PCR.

## Introduction

Classical swine fever (CSF), also known as ‘hog cholera’, is a highly contagious and often fatal disease of domestic pigs and wild boar [1]. The causative agent ‘CSF virus’ (CSFV), is a member of the Genus ‘*Pestivirus’* within the family ‘*Flaviviridae*’. The CSFV genome is a (+) ss RNA of about 12.3 kb in length, which contains untranslated regions at 5’ and 3’ ends and encodes a single polyprotein that is both co- and post-translationally processed to yield four structural (C, EO, E1 and E2) and 7-8 non-structural (Npro, p7, NS2, NS3, NS4A, NS4B, NS5A and non-structural protein 5B [NS5B]) viral proteins [2,3].

CSF, classified under list-A diseases by OIE, is considered as a trans-boundary animal disease by Food and Agriculture Organization [4]. It causes significant economic losses to the swine industry throughout the world. It is endemic in most of continental Western Europe, South America and Far East. In India, outbreaks of CSF have been reported from Uttar Pradesh, Maharashtra, Tamil Nadu and Punjab [5,6]. In the North Eastern region also, the outbreaks were reported from Nagaland, Mizoram and Assam [7-9].

The North East India contains 30% of the total pig population of India and pig husbandry plays an important role in the socio-economic development of this region, including the state of Mizoram. Department of Veterinary and Animal Husbandry, Government of Mizoram, has identified the CSF as the disease of special economic importance for Mizoram in their Annual Report, 2002-2003. Unrestricted movement of pigs from one region to another within the country and across the porous international border surrounding the North Eastern States, unavailability of adequate doses of vaccine, lack of timely diagnosis of disease, and poor public awareness are some of the important factors that might have helped in perpetuating the disease in the state. CSF thus demands special attention in North East India to minimize the losses suffered by poor farmers in remote rural areas and small farmers in semi-urban areas, who adopt pig farming as a subsidiary vocation to supplement their livelihood.

The present study was based on clinico-pathological investigation and molecular diagnosis of classical swine fever in pigs of Mizoram from 6 suspected outbreaks of the disease during August 2010 to July 2011.

## Materials and Methods

### Ethical approval

The work has been approved by the Institutional Animal Ethics Committee of the college.

### Sample collection

Pigs reared under organized and unorganized farms in 8 districts of Mizoram were regularly monitored for the occurrence of classical swine fever during the study period. Clinical changes shown by the affected pigs were recorded, and pigs died of suspected CSF infection were subjected to post-mortem examination. Various changes in the organs like kidneys, lymph nodes, intestine, spleen, tonsils, brain, heart, liver and lungs were recorded. The representative tissue samples were collected and transferred on ice and preserved at -80°C for molecular analysis. The tissue specimens were also preserved in 10% buffered formalin for histopathological examination.

### Histopathological examination of tissues

After proper fixation, the tissues were processed, and sections of 4-5 µm thickness were made and stained with routine hematoxylin and eosin for histopathological examination [10]. Sections were observed under Trinocular research microscope to record the pathological changes.

### RNA extraction and reverse transcription polymerase chain reaction (RT-PCR)

Total RNA from the collected tissue samples were extracted separately using TRIzol (Sigma, USA) reagent as per the manufacturer’s protocol. The purity and quantitation of extracted RNA were checked by spectrophotometric method (Biophotometer, Eppendorf, Germany). RT reactions were performed using random primer with the cDNA synthesis kit (Fermentas) as per the instructions given by the manufacturer. The 5´NTR [11], NS5B [12], E^rns^ [13] and E2 [14] glycoprotein encoding gene regions of CSFV were amplified by PCR using specific oligonucleotide primers (Table-1). PCR was performed in 25 µl reaction volume containing Dream Taq™ Buffer (×10), 200 µM of each dNTPs, 20 pM of each primer, 1.25 units of Dream Taq™ DNA polymerase and 0.5 µg of cDNA. The reactions were performed with cyclic conditions of 95°C × 2 min, 95°C × 1 min, optimal annealing temperature (Table-1), 72°C × 1 min and 72°C × 5 min.

**Table 1:** List of oligonucleotide primers used in the present study

Primer identity	Sequence	Expected product size	Ref no.	Annealing temperature applied
NTR-F	5’-CTA GCC ATG CCC WYA GTA GG 3’	421 bp	11	50°C
NTR-R	5’-CAG CTT CAR YGT TGA TTG T-3’			
E2-F	5’-AGR CCA GAC TGG TGG CCN TAY GA-3’	671 bp	14	55°C
E2-R	5’-TTY ACC ACT TCT GTT CTC A-3’			
E2-IF	5×TCR WCA ACC AAY GAG ATA GGG 3×	271bp	14	55°C
E2-IR	5×CAC AGY CCR AAY CCR AAG TCA TC 3×			
NS5B-F	5’-GAC ACT AGY GCA GGC AAY AG-3’	449 bp	12	56°C
NS5B-R	5’-AGT GGG TTC CAG GAR TAC AT-3			
ERNS-F	5’-CATGCCATGGCCCTGTTGGCTTGGGCGGTGATA-3’	735bp	13	65°C
ERNS-R	5’-GGAATTCTCAGGCATAGGCACCAAACCAGG-3’			

For E2 inner fragment (271bp), a nested PCR was performed using the PCR product of the outer E2 fragment (671bp) as template DNA with same thermal profile.

Amplified products were separated by agarose gel electrophoresis (1.5% agarose in ×0.5 Tris-borate-ethylenediaminetetraacetic acid) at 5 V/cm for 2 h and stained with ethidium bromide (0.5 µg/ml). A standard molecular marker (100 bp DNA ladder) was included in each gel. DNA fragments were observed by ultraviolet transilluminator and photographed by gel documentation system (BioRad, Germany).

## Results

### Clinical case reports of the disease outbreak

**Figure-1:**
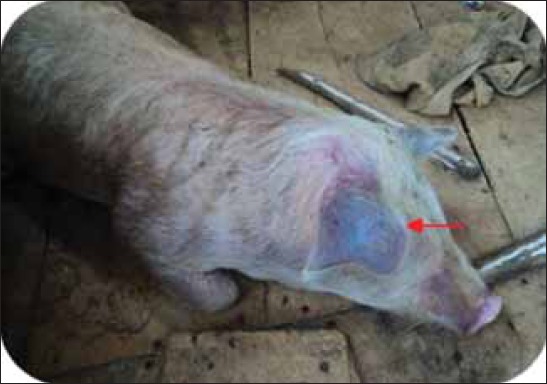
Purple discoloration of skin at the tip of the ears in pigs suffered from acute classical swine fever

A total of 31 clinically affected pigs suspected for CSF and which were subsequently died, from 6 different districts of Mizoram were included in the study. Clinical examination of the affected pigs showed severe depression, complete anorexia and high rise of body temperature (103-106°F). Pigs were huddled together in the corner of the house, exhibited labored breathing, staggering gait or swaying movement of the hindquarters and erythematous lesions in the abdominal region, ears and in the medial side of the legs (Figure-1). Conjunctivitis, thick ocular discharge and watery nasal discharge were also observed (Table-2).

**Table 2:** Clinical score and histopathological score of the affected pigs studied

Necropsy no.	Clinical score (0=3 scale: normal0, mild1, moderate2, severe3)								Histopathology (0=3 scale: normal0, mild1, moderate2, severe3)						RTPCR result (tonsiland spleen tissue)
	
	Dullness	Anorexia	Conjunctivitis	Nasal discharge	Skin (petechie, haemorrhage)	Respiratory sign	Fever	Total	Lungs	Lymph nodes	Spleen	Tonsil	Kidney	Liver	
D-1	3	3	2	2	3	3	3	19	3	3	3	3	3	3	+Ve
D-2	3	3	1	2	2	3	3	17	3	3	3	3	3	2	+Ve
D-3	2	2	0	1	1	2	2	10	2	1	2	1	2	1	+Ve
D-4	2	2	0	1	1	2	2	10	2	1	2	1	1	1	+Ve
D-5	3	3	1	2	3	3	3	18	3	3	3	2	2	2	+Ve
D-6	3	3	0	2	2	3	3	16	3	2	3	2	2	2	+Ve
D-7	3	3	1	2	3	3	3	18	3	2	3	2	3	2	+Ve
D-8	3	3	1	2	2	3	3	17	3	3	3	2	2	2	+Ve
D-9	2	2	0	1	1	2	2	10	3	2	2	1	2	2	+Ve
D-10	3	3	1	2	3	3	3	18	3	3	3	3	3	2	+Ve
D-11	3	3	1	3	2	3	3	18	3	3	3	3	3	2	+Ve
D-12	3	3	0	2	2	3	3	16	3	2	3	2	2	2	+Ve
D-13	3	3	1	2	2	3	3	17	3	3	3	2	3	2	+Ve
D-14	2	2	0	1	1	2	2	10	2	2	2	1	2	1	+Ve
D-15	3	3	1	1	1	2	3	14	3	2	3	2	2	2	+Ve
D-16	2	2	0	0	1	2	2	9	2	2	2	1	1	1	+Ve
D-17	3	3	1	2	3	3	3	18	3	3	3	3	3	2	+Ve
D-18	3	3	2	2	3	3	3	19	3	3	3	3	3	3	+Ve
D-19	3	3	1	1	2	3	3	16	3	3	3	3	2	2	+Ve
D-20	3	3	1	2	3	3	3	18	3	3	3	3	2	3	+Ve
D-21	2	2	0	0	1	2	2	9	2	2	2	2	1	1	+Ve
D-22	3	3	1	2	1	3	3	16	3	2	3	2	2	2	+Ve
D-23	3	3	1	2	1	3	3	16	3	2	3	2	2	2	+Ve
D-24	2	3	1	1	1	3	3	14	3	2	3	2	2	2	+Ve
D-25	2	3	0	1	1	2	2	11	2	2	2	1	1	1	+Ve
D-26	3	3	1	2	3	3	3	18	3	3	3	2	2	3	+Ve
D-27	3	3	2	2	3	3	3	19	3	3	3	3	3	3	+Ve
D-28	3	3	1	1	3	3	3	17	3	3	3	2	2	2	+Ve
D-29	2	3	0	0	1	2	3	11	2	2	2	1	1	1	+Ve
D-30	3	3	0	2	2	3	3	16	2	2	3	2	2	2	+Ve
D-31	3	3	1	2	2	3	3	17	3	3	3	3	2	2	+Ve

RTPCR=Reverse transcription polymerase chain reaction

### Post-mortem examination of the dead animals

Post-mortem examination of dead pigs showed multiple petechiation or purple discoloration of the skin in the abdominal region, ears and in the medial side of legs. Opening of the carcasses revealed subcutaneous ecchymotic hemorrhages. All the superficial lymph nodes were swollen, edematous, hemorrhagic and dark tan colored in appearance. Typical infarction in the spleen was also observed. The spleen was enlarged and congested with small raised hemorrhagic areas. Mesenteric blood vessels were severely congested. Petechial hemorrhages on the epicardial surface of the heart were also noticed. Liver was dark, enlarged and congested in all cases. Non collapsing, hemorrhagic lungs with pneumonic areas were also observed. Pinpoint or petechial hemorrhages in the sub capsular region of the kidneys resembling ‘Turkey egg kidney’ were also observed. Cross section of kidneys revealed severe congestion in the cortico-medullary junction.

### Histopathological examination of tissues

**Figure-2:**
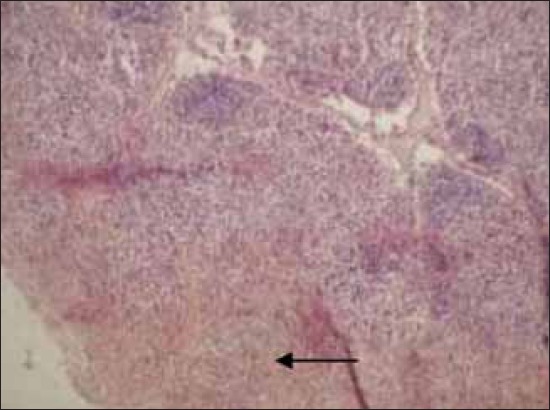
Diffuse hemorrhages in the cortex of mesenteric lump node. H and E, ×100

**Figure-3:**
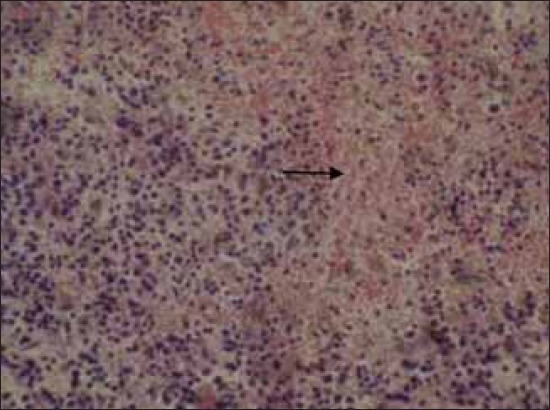
Diffuse hemorrhages throughout the splenic parenchyma Spleen. H and E, ×200

**Figure-4:**
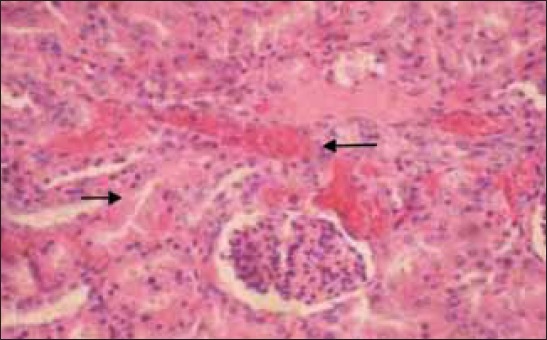
Hemorrhages and tubular degeneration in the cortex of kidney. H and E, ×200

**Figure 5:**
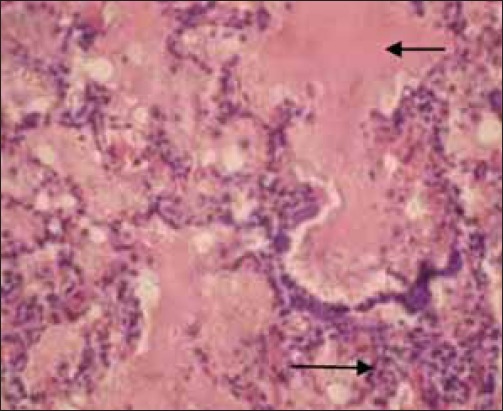
Interstitial pneumonia with serofibrinous exudates in alveolar space in lungs. H and E, ×400

**Figure-6:**
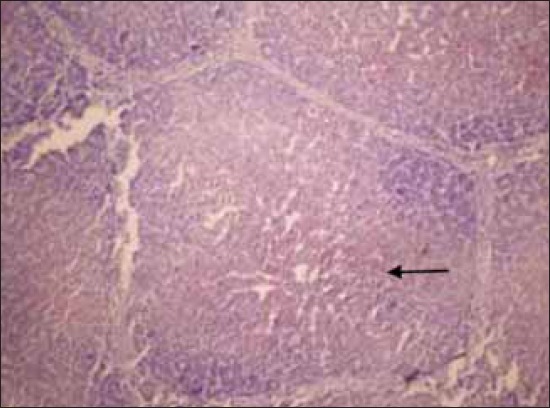
Centrilobular congestion and hepatocellular degeneration. H and E ×100

The histopathological examination of lymph nodes and tonsils showed congestion of the blood vessels along with focal to diffuse areas of hemorrhage. The lymphoid follicles were depleted and showed necrotic cellular debris and hemorrhages (Figure-2). Focal to diffuse hemorrhages in the red pulp and depletion of the lymphocyte in the splenic corpuscles were observed in the spleen (Figure-3). Kidneys showed congestion of the blood vessels and focal areas of hemorrhage in the cortico-medullary regions and interstitial nephritis characterized by infiltration of mononuclear cells in the interstitial spaces (Figure-4). Focal areas of acute glomerular nephritis characterized by swelling of the glomerular tuft expanding up to the Bowman’s space with cellular infiltration and hemorrhages were also observed. Tubular epithelium showed degeneration and necrosis. Lungs showed lesions of interstitial pneumonia with extensive hemorrhage and serofibrinous exudates in the air spaces (Figure-5). The inter-alveolar septa were thickened with infiltration of mononuclear cells and congestion of alveolar capillaries. The alveolar space, bronchi and bronchioles were filled with serofibrinous exudate. At places, peribronchiolar areas showed hyperplasia of lymphoid tissue with the formation of lymphoid aggregates. Histopathological changes in the liver were characterized by congestion of central vein and sinusoidal spaces. The hepatocytes showed centrilobular degenerative changes with focal areas of necrosis (Figure-6). The peyer’s patches in the ileum showed depletion of lymphocytes in the lymphoid follicles.

### RT-PCR based detection of CSFV specific genes

**Figure-7:**
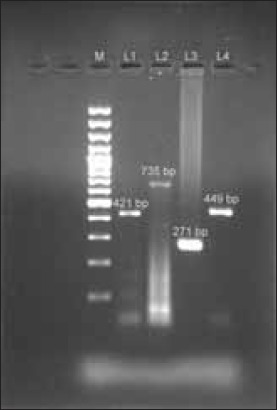
1.5% Agarose gel electrophoresis showing amplified product of L1-5‘NCR (421 bp), L2-E^rns^ (735 bp), L3- E2 (271 bp) and L4- NS5B (449 bp) gene fragments

In the present study, all the tissue samples (tonsil, lymph nodes, spleen) from 31suspected cases revealed positive results for CSFV by RT-PCR with the amplified PCR product of 421bp (5´NCR), 271bp (E2), 449bp (NS5B) and 735bp (E^rns^) (Figure-7).

## Discussion

All the suspected animals under the study exhibited typical clinical signs of CSF as recorded by other workers from India and abroad [7-9,15-17]. However, depending upon the virulence and pathogenicity of the viral strain, there could be variability in the clinical picture of the disease. The highly virulent strains kill nearly all pigs, the moderate virulent strains cause sub- acute illness in post-natally infected piglets and abnormalities in porcine foetuses, and avirulent strains are virtually non-pathogenic for both pigs and foetuses. In addition, the host factors, like age, immune status and breed of affected pigs may also influence the clinical form of the disease [18,19]. In the present study, the affected animals showed acute course of the disease and died within 10 days after appearance of clinical signs, which is indicative of involvement of a highly virulent CSFV strain.

Post mortem lesions of the dead pigs also indicated an acute form of CSF primarily due to severe vascular alterations in various organs leading to hemorrhagic diathesis, which produces hemorrhages in kidney, lymph nodes, skin, mucous and serous membranes, as well as spleen infarctions. Our results are also in corroboration with other workers [8,9,15]. The histopathological observations were also found similar to those described by other workers [8,9]. Although, the microscopic lesions of CSF are well-defined, it is necessary to characterize the derangement caused by each field isolate to determine factors such as tissue tropism and distribution.

The RT-PCR used for detection and confirmation of CSFV in tissue samples from tonsil, lymph nodes and spleen targeting the 5´NCR (421bp), E2 (271bp), NS5B (449bp) and E^rns^ (735 bp) gene fragments, amplified the expected products and confirmed the diagnosis of CSF in pig population of Mizoram. The diagnosis of CSF is generally confirmed by PCR targeting relatively conserved genes region. The 5´NTR nucleotide sequence is highly conserved among all members within the genus *Pestivirus*, thus it is very useful for the characterization of species or genotype. The envelope of CSFV contains three glycoprotein E^rns^, E1 and E2 [20]. E2 is the major glycoprotein, which is highly immunogenic and induces high virus neutralizing titers of serum antibodies [21]. The CSFV NS5B encodes an RNA- dependent RNA polymerase (RdRp) with 70-75% identical amino acid sequences. However, it appears to have conserved tertiary structure rather than primary sequence that makes it a valid candidate to CSFV and non-CSFV *Pestivirus* differentiation [22].

## Conclusion

Classical Swine Fever is found to be highly endemic disease in Mizoram and the viral strains circulating in this region are highly virulent. The disease can be specifically diagnosed by RT-PCR.

## Author Contributions

DM: Collection and processing of samples. T.KR: Planning of research methodology, technical support. RC: Overall supervision of the work, editing of the manuscript. T.KD: Preparation of samples, PCR, preparation of the manuscript. All authors read and approved the final manuscript.

## Computing Interests

The authors declared that there is no conflict of interest.
